# Evaluating the Use of Multilocus Variable Number Tandem Repeat Analysis of Shiga Toxin-Producing *Escherichia coli* O157 as a Routine Public Health Tool in England

**DOI:** 10.1371/journal.pone.0085901

**Published:** 2014-01-17

**Authors:** Lisa Byrne, Richard Elson, Timothy J. Dallman, Neil Perry, Philip Ashton, John Wain, Goutam K. Adak, Kathie A. Grant, Claire Jenkins

**Affiliations:** 1 Gastrointestinal Emerging and Zoonotic Infections Department, Health Protection Services, Public Health England, Centre for Infectious Disease Surveillance and Control, London, United Kingdom; 2 Gastrointestinal Bacteria Reference Unit, Microbiology Services, GBRU, Public Health England, Centre for Infectious Disease Surveillance and Control, London, United Kingdom; 3 Faculty of Medicine and Health Services, Norwich Medical School, University of East Anglia, Norwich, United Kingdom; U. S. Salinity Lab, United States of America

## Abstract

Multilocus variable number tandem repeat analysis (MLVA) provides microbiological support for investigations of clusters of cases of infection with Shiga toxin-producing *E. coli* (STEC) O157. All confirmed STEC O157 isolated in England and submitted to the Gastrointestinal Bacteria Reference Unit (GBRU) during a six month period were typed using MLVA, with the aim of assessing the impact of this approach on epidemiological investigations. Of 539 cases investigated, 341 (76%) had unique (>2 single locus variants) MLVA profiles, 12% of profiles occurred more than once due to known household transmission and 12% of profiles occurred as part of 41 clusters, 21 of which were previously identified through routine public health investigation of cases. The remaining 20 clusters were not previously detected and STEC enhanced surveillance data for associated cases were retrospectively reviewed for epidemiological links including shared exposures, geography and/or time. Additional evidence of a link between cases was found in twelve clusters. Compared to phage typing, the number of sporadic cases was reduced from 69% to 41% and the diversity index for MLVA was 0.996 versus 0.782 for phage typing. Using MLVA generates more data on the spatial and temporal dispersion of cases, better defining the epidemiology of STEC infection than phage typing. The increased detection of clusters through MLVA typing highlights the challenges to health protection practices, providing a forerunner to the advent of whole genome sequencing as a diagnostic tool.

## Introduction

Shiga toxin-producing *Escherichia coli* (STEC) are associated with human illness and are defined by the presence of the phage-encoded Shiga toxin genes, *stx1* and/or *stx2*. Symptoms of STEC infection range from mild gastroenteritis through to severe bloody diarrhoea and approximately 6% of cases develop haemolytic uraemic syndrome (HUS);[1] HUS is a serious condition where shiga toxins affect the blood and kidneys. It most frequently affects children and is recognised as the most common cause of kidney failure in children.

The main reservoir of STEC in England is cattle although it is carried by other animals, mainly ruminants.[2] Transmission to humans occurs through direct or indirect contact with animals or their environment, consumption of contaminated food or water and through person-to-person contact.[3]–[6] STEC may cause both sporadic and epidemic infections and several large outbreaks have been recorded.[7]–[9]Traditionally, outbreaks of STEC are identified through routine investigation of cases, either through identifying common features between cases in terms of exposures, the appearance of microbiological subtypes (i.e. serogroups and phage types) among cases which are temporally or geographically linked, or indications that the number of cases in a particular location or of a particular subtype is higher than expected. Between 2009 and 2012, there were 67 reported outbreaks, affecting 737 individuals in England and virtually all of these were identified following investigation either locally by the Public Health England Centre (PHEC) or nationally by the Department of Gastrointestinal, Emerging and Zoonotic Infections (GEZI) (unpublished data). The remaining 2,982 cases not attributed to outbreaks were either sporadic cases or due to person-to-person transmission within households.

Each year, over 1000 isolates of presumptive *E. coli* O157 (the most frequently detected serogroup in England) are submitted to the Gastrointestinal Bacteria Reference Unit (GBRU), (the national reference laboratory for gastrointestinal pathogens in England) and on average approximately 900 are confirmed as STEC O157. All confirmed STEC O157 isolates are phage typed,[10] and the most frequently reported phage types each year are PT 21/28 and PT 8. Standard Enhanced Surveillance Questionnaires (ESQ) data are routinely collected from presumptive cases of STEC, often before isolates are confirmed as STEC by the GBRU. These data are scrutinised and identification of common features between cases is most often the first indication of an outbreak. Where enhanced surveillance data identifies cases which may be linked, strains are selected for additional molecular typing by multilocus variable number tandem repeat (VNTR) analysis (MLVA).[11] While routine phage typing provides a rapid, robust and cost effective screen, MLVA, provides a more discriminatory, portable typing technique. MLVA thus provides more sensitive microbiological support for epidemiological links and, since implementation of its selective use in 2006, has made a valuable contribution to outbreak investigations in England and elsewhere. [12]; [13]However, MLVA typing of all isolates has resource implications because of the high numbers of isolates received by GBRU during peak periods.

From the 1^st^ May 2012, all confirmed STEC O157 isolates submitted to GBRU were typed using MLVA, with the aim of evaluating this typing approach for prospective outbreak identification and assessing how it would impact on epidemiological investigations. Data and findings from the first six months of this strategy are presented herein.

## Materials and Methods

### Microbiological characterisation

Detection and confirmation of STEC at GBRU included biochemical identification and serotyping of bacterial isolates. Real time polymerase chain reaction (PCR)was performed to determine the presence of Shiga toxin genes *stx1* and/or *stx2*.[14] Strains belonging to serogroup O157 were further differentiated by phage typing.[10]

From 1^st^ May 2012, all STEC O157 strains submitted to GBRU were typed using MLVA. Amplification of eight MLVA loci was performed in 20 ul reactions volumes on all isolates in two quadruplex PCR reactions. [11]; [15] VNTR-10 locus was not tested. The method was modified to use the following dye labels in the forward primers in the amplifications: NED in VNTR3 and VNTR17; 6-FAM in VNTR34, VNTR19, VNTR9 and VNTR36 and VIC in VNTR25 and VNTR37. Sizing of the amplified products was on an ABI 3730 Genetic Analyzer with 600 LIZ (Applied Biosystems) as size standard and data were analysed with Peakscanner software. Fragment sizes were imported into Bionumerics software via an algorithm that calculated the tandem repeat numbers for each locus. A minimum spanning tree was constructed to visually compare MLVA profiles with phage types.

### Enhanced surveillance of STEC in England

Local laboratories report presumptive isolates of STEC directly to PHE Centres, who arrange for an ESQ to be administered to patients either by local health protection practitioners or environmental health professionals. The ESQ collects demographic details; risk status; clinical condition (including progression to HUS); household or other close contact details; laboratory results; exposures including travel, food and water consumption, contact with animals and environmental factors; epidemiological case classification; and outbreak/cluster status. Completed questionnaires are forwarded for inclusion in the National Enhanced Surveillance System for STEC in England (NESSS) which is managed by the PHE GEZI department.

### Case categorisation and comparison

#### Case Definitions

Domestic case: An STEC case with no travel outside of the UK reported in the seven days prior to onset of illness.

Travel case: An STEC case with travel outside of the UK reported in the seven days prior to onset of illness.

Household case: An STEC case epidemiologically and microbiologically linked to one or more cases in the same household.

Cluster case: An STEC case epidemiologically and microbiologically linked to one or more case outside of the same household.

Sporadic case: An STEC case with no known links to any other STEC cases.

Case definitions were applied first on the basis of ESQ data and results of standard microbiological methods (i.e. phage typing/*stx* profiles). Cases were re-categorised on the basis of MLVA results. Comparisons were made between case categories based on phage typing and MLVA. The discriminatory ability of both phage typing and MLVA typing were calculated using Simpson's index of diversity as previously described.[16]

#### Categorisation and evaluation of clusters

Clusters were grouped into three categories:

- Household clusters
- Known Clusters- detected through routine public health follow-up of STEC cases identifying linked cases
- MLVA identified clusters – newly identified through MLVA typing

For clusters newly identified through MLVA, the ESQ's for cases were retrospectively reviewed for epidemiological links including:

- Temporal linkage: Cases with onset of illness occurring within seven days of each other.
- Exposure linkage: Cases reporting shared exposures, including geographical links (all cases resided or visited a location within one PHE region), as reported on the ESQ.

## Results

### Case categorisation and comparison

**Figure 1:**
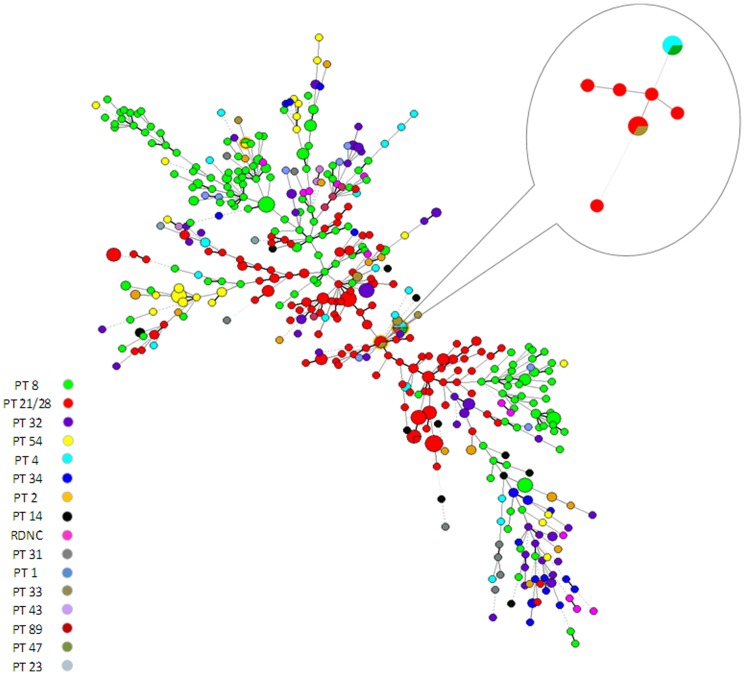
Minimum spanning tree of STEC O157 isolates MLVA profiles categorised by phage type^1^. 1. Includes 539 confirmed cases of STEC O157 in England. The size of nodes is proportional to the number of instances of that unique profile. Join lines represent locus variants: Related SLV's and DLV's are represented through a solid line while >two locus variants are denoted through a dotted join.In three clusters MLVA profiles spanned two phage types, these are highlighted in the figure. Inset presents two of these instances where the same MLVA profile was reported in two different phage types.

Between 1^st^ May and 31^st^ October 2012, 556 confirmed cases of STEC O157 in England were reported and 16 different PT's were identified amongst cases; the most frequently reported PT overall was PT 8 (n = 189, 33.9%), followed by PT 21/28 (n = 160, 28.8%). STEC O157 isolates from 539 cases (96.9%) were further typed by MLVA and a total of 341 unique (>2 SLV's) MLVA profiles were identified. Over three-quarters (n = 258) of profiles were unique to one case. Of the remaining profiles (24%), half (n = 41) occurred more than once due to known household transmission and half (n = 42) occurred more than once outside of households (i.e. community clusters). The concurrence between MLVA profiles and PT was high, with just three instances where the same MLVA profile covered more than one PT (figure 1). Simpson's diversity index for the phage typed isolates was 0.782, and for MLVA typing was 0.996, indicating the increased discriminatory power of this method.

**Figure 2:**
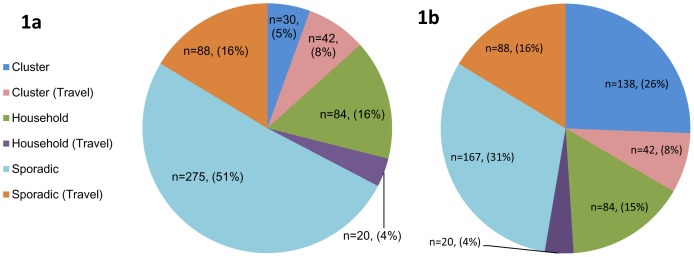
a) Case classification of STEC O157 cases categorised through VESQ data and phage typing, and 2b) Case classification of STEC O157 cases categorised through VESQ data and MLVA typing: May-October 2012^1,2^. 1.Includes 539/556 confirmed cases who had isolated typed by MLVA. 2.Travel cases reported travel outside of the UK in the seven days prior to onset of illness

ESQ data indicated a history of foreign travel for 150 cases (27.8%), including 20 cases comprising eight household clusters and 42 cases linked to 13 travel associated clusters (Figure 2a). Among 389 domestic cases, eight community clusters comprising 37 (8.9%) cases and 34 household clusters comprising 86 (22.1%) cases were reported. The remaining 267 cases (68.6%) were not linked to any known clusters and were classed as sporadic through ESQ data and phage typing. However, unique MLVA profiles were reported for less than half of domestic cases (42.9%, n = 167) and 101 were re-categorised as community cluster cases (Figure 2b). MLVA typing indicated 28 separate non-travel community clusters: 20 newly identified through MLVA typing. Compared to phage typing, the proportion of domestic sporadic cases was thus reduced by 27.8% through MLVA.

### Cluster categorisation and evaluation

#### (i) Household clusters

A fifth (n = 106) of all cases comprised 42 different household clusters, eight of which were travel-related. Household clusters ranged between two and seven cases in size, with a median of 2 cases (IQR: 2, 3). Of the 42 clusters, in 24 households all cases had the same MLVA profile, 11 exhibited Single locus variants (SLVs), five double locus variants (DLVs) and two had >2 locus variants.

#### (ii) Known Clusters

Twenty-one of the 41 community clusters defined through MLVA typing were known clusters, previously identified as epidemiologically linked through surveillance data from ESQ's and phage typing. This included 13 clusters of cases associated with travel to Malta (3), Turkey (3), Cape Verde (2), Tunisia (1), Morocco (1), Egypt (1), Spain (1) and Israel (1). Eight non-travel community clusters comprising 37 cases (Table 1) were detected through routine follow-up of cases and were investigated. Exposures included attendance at nurseries and schools (4), recent consumption of minced beef products outside the home (1), contact with ruminants in a park or farm (2) and attendance at a wedding (no food identified) (1). All eight clusters comprised cases which were temporally and geographically linked (all cases resided or visited a location within one PHEC).Inclusion of cases was informed through MLVA typing in all eight clusters. One cluster of PT 21/28 *stx2* was first identified when three cases reported consuming beef burgers at the same food outlet. Investigations initially focused on the food outlet, however, six additional cases not linked to this food outlet were identified by GBRU as having the same MLVA profile and investigations were widened. Raw minced beef contaminated with STEC O157 during the supply chain was suspected as the source of infection.

**Table 1:** Summary of community clusters identified through routine follow-up of cases: May- October 2012.

Cluster	PT/*stx*	Total cases	Cases identified through MLVA^1^
1. minced beef	PT 21/28 *stx*2	9	6
2. wedding reception	PT 8 *stx*1+2	2	0
3. nursery/school A^2^	PT 4/47 *stx*2	2	0
4. nursery/school B	PT 21/28 *stx*2	6	0
5. nursery/school C	PT 8 *stx*1+2	4	0
6. nursery/school D	PT 32 *stx*2	5	0
7. Petting farm^2^	PT 4 *stx*2	2	0
8. Country park	PT 54 *stx*2	8	0
Total		38	6

^1^ Cases not linked with recognised clusters through phage typing and VESQ data.

^2^ One case from the farm seeded the outbreak in nursery A.

MLVA did not identify additional cases in the six other clusters. However, in Cluster#3 (Nursery cluster A), MLVA indicated that despite different PT's (figure 1), the cases were linked and that the cluster was seeded by a case who was part of a separate cluster associated with visiting a farm (Cluster #6). The outbreak associated with exposure to animals in a country park comprised strains with MLVA profiles that had DLVs, whereas the other six outbreaks were comprised of strains that shared identical profiles or were SLVs.

#### (iii) MLVA identified clusters

An additional 20 community clusters not detected through ESQ data and phage typing were identified through MLVA. These clusters ranged in size between two and 12 cases, with a median of three cases (IQR: 3, 7). Eighteen of the 20 clusters (92 cases) had associated strains which were either PT 21/28 *stx*2 or PT8 *stx*1+2. ESQ data for cases from the clusters were reviewed for links between cases, and epidemiological links between cases were identified in twelve clusters (Table 2).

**Table 2:** Summary of evidence from NESSS for linkage between cases for community clusters of STEC O157 identified through MLVA between 1^st^ May and 31^st^ October 2012.

Evidence of linkage between cases	No. clusters	Total No. cases	Min. cases/cluster	Max. cases/cluster
MLVA, temporal^1^ & shared exposures^2^	4	29	2	12
MLVA & temporally related cases^1^	8	45	2	11
MLVA linked only	8	27	2	7
All clusters	20	101	2	13

^1^ Cases with onset dates between zero and seven days apart.

^2^ Includes residing in or travel within the UK to the same area, shared direct or indirect contact with animals and/or their environment.

#### Clusters with temporal and exposures linkage

Four MLVA clusters had cases both occurring close together in time and additional evidence of linkage between cases. Definitive sources of infection were not identified but cases were linked geographically and/or through exposures: Three of the clusters consisted of cases from different English regions who had travelled to the same areas on holiday and some had visited the same attractions or undertaken the same activities. The fourth cluster was of two cases who lived in the same town. One cluster of two in a household comprised cases with an identical MLVA proflle but one case had a PT54 strain and the other PT8.

One MLVA cluster of seven cases comprised cases from different regions in England. Reviewing the ESQ data revealed that two cases had visited the same village, where another case was resident. Four cases were re-interviewed and all reported visiting the same public house. There were no common consumption patterns between the cases, and two cases had visited the premises outside of the seven day exposure period. The potential source could have been a person or the environment or it may that contamination was taken into the public house. Three other cases did not report travel to the same location and no obvious shared exposures were reported on the ESQ. Because of the time that had elapsed between notification and onset no further follow-up of these cases was undertaken.

#### Clusters with temporal linkage only

Eight MLVA clusters consisted of 45 cases with onset dates close together in time (maximum seven days between cases) but no common geography or exposures were reported on the ESQ's. Five clusters each had two to three cases, but one larger cluster comprised eight cases (one with a different PT, figure 1), and two clusters 11 cases each. The epidemic curves, lack of unusual exposures and the wide geographical distribution of cases were suggestive of widely distributed food-borne sources of infection. For one cluster of 11, nine cases were re-interviewed. The only common exposure among all nine cases was the consumption of pre-packed salad from different branches of one major supermarket chain. No further cases were reported and it was not possible to undertake further analytical epidemiology.

#### Clusters with no additional linkage

No additional evidence for a cluster could be found from the ESQ for eight MLVA clusters, totalling 27 cases. The size of these clusters varied from two to seven cases. Within these clusters, cases did not appear to be linked geographically, were dispersed over time, spanning several months, and no obvious shared exposures were reported on the ESQ's.

## Discussion

A number of studies have described the ease of use and timeliness of MLVA and demonstrated the discriminatory power of this approach over phage typing and pulse-field gel electrophoresis.[17]-[19] This study also clearly demonstrates the increased discriminatory power of MLVA over phage typing, with three-quarters of MLVA profiles being unique to one case. The addition of MLVA typing identified twenty additional clusters among domestic cases, reducing the proportion of sporadic cases by almost 30%. This approach thus better defines the epidemiology of STEC infection in England, providing a more accurate description of sporadic and cluster cases. Analysis of more accurately defined sporadic cases over time will facilitate better characterisation of the population at risk and enable a more accurate description of the important exposures that may be leading to these infections.

In England, the most prevalent STEC O157 PT's are PT21/28 and PT8. Five of the eight known clusters identified through public health investigation and supplemented by phage typing, were of a PT other than PT 21/28 or PT 8. Detection of rarer PT's facilitates identification of clusters. In addition, all but one of the known clusters were linked to a precise location, and all cluster cases were temporally linked. In contrast, only a fifth of the MLVA identified clusters were linked geographically, and not to precise locations. In addition, most were of PT 21/28 or PT8, as per all STEC O157 cases, making it extremely difficult to detect such clusters with phage typing alone. There may be under-reporting of PT 8 cases as symptoms appear less severe which may increase the likelihood of PT8 clusters occurring undetected. In contrast, PT 21/28 is associated with more severe disease and progression to HUS than other PT's. [1] As the greatest burden of domestic infection is attributed to this PT, it is important to detect clusters, identify sources of infection and establish control measures.

MLVA was useful in informing investigation of known clusters. The additional data can be used to elucidate previously undisclosed links, such as in the farm cluster seeding the nursery cluster, thus highlighting gaps in control measures. MLVA data had a substantial impact on investigations of Cluster#1 (associated with minced beef outbreak), where it captured additional cases and widened the focus of the outbreak control team. Through increased ascertainment of linked cases, both the likelihood and accuracy of determining the source of infection was improved.

Although MLVA identified additional clusters, analyses of data did not lead to an increased chance of a public health intervention. For most of the 20 clusters newly detected through MLVA, reviewing the ESQ data revealed temporal clustering of cases, but additional evidence of shared exposures was indicated in just four clusters. In such clusters where cases are linked to a relatively broad geographic area and the potential source of infection is environmental, it is difficult to pinpoint the source of infection from ESQ data. For eight clusters, no temporal, geographic or exposure linkage was detected. The ability to establish epidemiological links between cases is in part diminished due to the small sample size of the clusters, making it difficult to confidently establish commonality of exposures. ESQ data were retrospectively reviewed when some time had passed since most cases were ill, the clusters were small in size and were temporally contained. Therefore the ability to undertake further analytical study was limited.

There were three larger clusters comprising nationally distributed cases with apparent temporal links suggestive of a food-borne source of infection. While, the ESQ, introduced in England in 2009, has made a major contribution to STEC surveillance, it was not designed to capture the highly detailed information often required to definitively link cases to a shared exposure. The ESQ is not a trawling questionnaire so the vehicle of infection, for example, could be a food-stuff not included in the questionnaire or included but not in enough detail to identify it. Additionally, similarities in consumption between cases may reflective of common population behaviours, such as in the cluster of cases reporting consumption of pre-packed salad.

While this study demonstrated the added value of MLVA in increasing recognition of clusters and providing insight into the microbiology and epidemiology of STEC O157 in England, it raises questions in terms of how to respond in terms of public health. Most STEC clusters are small so the chance of proof of source and hence intervention is reduced, while analytical studies are not possible. The resource involved in investigating clusters is not inconsiderable; input from a wide range of multi-disciplinary professionals is required and in nationally dispersed clusters, co-ordinating local investigations is challenging. In real-time surveillance the eventual size of a cluster is unknown, and difficulties lie in deciding at what point hypothesis generation exercises should be initiated. The delay in obtaining MLVA data (although MLVA is more rapid than PFGE, the MLVA data is often available more than eight days after the patient becomes symptomatic), and thereby identifying linked cases, may also impede investigation. The increased detection of clusters through the additional sensitivity of MLVA typing provides a forerunner for the advent of next generation sequencing of clinical isolates and highlights both the advantages to, and the challenges in public health practice, in utilising molecular methods as routine, which need to be addressed.

PFGE remains the method of choice for outbreak investigation in many countries, although the use of MLVA is increasing as the contribution of the MLVA approach to overall surveillance of STEC O157, in addition to outbreak investigation, is recognised. Despite the challenges described above, the universal MLVA approach has revealed a more accurate picture of the extent of linked cases in England and demonstrated that while STEC O157 clusters in England are small, they contribute a significant proportion of cases. Continuation of MLVA typing on all confirmed STEC O157 isolates submitted to the GBRU will generate more data on the spatial and temporal dispersion of cases of STEC O157. Furthermore, in conjunction with enhanced surveillance data and timely public health investigation of clusters, MLVA offers the potential to provide a more accurate measure of clusters, insight into routes of this infection in England and evidence to inform health protection responses at local and national levels.
